# A prospective randomized crossover trial investigating melatonin versus sleep deprivation for sleep induction in nap electroencephalography

**DOI:** 10.1002/epi4.70169

**Published:** 2025-11-10

**Authors:** Valentina De Giorgis, Costanza Varesio, Massimiliano Celario, Carlo Alberto Quaranta, Francesca Ferraro, Ludovica Pasca, Guido Fedele, Grazia Papalia, Michela Palmisani, Cinzia Fattore, Paola Rota, Valentina Franco

**Affiliations:** ^1^ Department of Brain and Behavioral Sciences University of Pavia Pavia Italy; ^2^ Department of Child Neurology and Psychiatry IRCCS Mondino Foundation Pavia Italy; ^3^ Full Member of ERN Epicare Pavia Italy; ^4^ AFI—Associazione Farmaceutici dell'Industria (AFI) Milan Italy; ^5^ Clinical and Experimental Pharmacology Unit, Department of Internal Medicine and Therapeutics University of Pavia Pavia Italy; ^6^ IRCCS Mondino Foundation Pavia Italy; ^7^ Department of Biomedical, Surgical and Dental Sciences University of Milan Milan Italy; ^8^ Institute for Molecular and Translational Cardiology (IMTC) Milan Italy

**Keywords:** 6‐hydroxy‐melatonin, epilepsy, melatonin, nap electroencephalography, sleep induction, sleep latency

## Abstract

**Objective:**

Electroencephalography (EEG) plays a fundamental role in the diagnosis and classification of epilepsy, and inducing sleep during EEG can improve patient cooperation and enhance the detection of epileptiform activity. Despite its importance, there is currently no standardized approach for sleep induction in pediatric EEG recordings. Consequently, practices such as melatonin administration and sleep deprivation are commonly utilized. This study aimed to compare the effectiveness of 5 mg melatonin versus partial sleep deprivation in inducing sleep during nap‐time EEGs in children with epilepsy.

**Methods:**

A randomized crossover trial was conducted involving 33 participants (mean age 14.5 years), each undergoing EEG following either melatonin administration or partial sleep deprivation. In the melatonin arm, participants received an oral dose 30 min before the recording, while in the sleep deprivation arm, sleep was restricted the previous night. The primary outcome was sleep onset latency, defined as the time from relaxation to non‐REM stage 2 sleep on EEG. Additionally, melatonin and its metabolite, 6‐hydroxy‐melatonin, were measured using liquid chromatography coupled with tandem mass spectrometry (LC–MS/MS).

**Results:**

The study showed a mean sleep onset latency of 8.5 min after sleep deprivation and 10.1 min after melatonin administration, with a mean difference of 1.5 min. The analysis of covariance conducted with stratification based on sleep onset latency (five classes) and considering all patients confirmed that melatonin is non‐inferior to sleep deprivation in sleep onset latency, with 97.5% lower confidence limits of −0.37. Melatonin levels in the treated group confirmed adequate absorption, while they were undetectable in the sleep‐deprived group.

**Significance:**

Melatonin is non‐inferior to partial sleep deprivation in reducing sleep onset latency, with comparable diagnostic yield and a favorable tolerability profile. The study demonstrates that 5 mg of melatonin is a safe, effective, and well‐tolerated alternative to partial sleep deprivation for sleep induction in pediatric EEG evaluations. Given its ease of use and consistent results, melatonin may be recommended as a practical standard for facilitating EEG recordings in children, particularly those with neurodevelopmental disorders.

**Plain Language Summary:**

This randomized crossover trial evaluated the effectiveness and tolerability of melatonin administered before an EEG recording to induce sleep, compared with partial sleep deprivation. The results showed that melatonin helped children achieve sleep and was non‐inferior to sleep deprivation. Moreover, melatonin demonstrated a favorable tolerability profile, representing a safe and easier alternative to support sleep during EEG, particularly in children with neurodevelopmental disorders, and could enhance the efficiency and quality of pediatric EEG recordings.

**Key points**

- The study compared the effectiveness of 5 mg melatonin versus partial sleep deprivation in inducing sleep during nap‐time EEG in children with epilepsy.
- A randomized crossover trial was conducted with 33 pediatric participants undergoing EEG after either melatonin administration or partial sleep deprivation.
- Sleep onset latency was comparable between melatonin (10.1 min) and sleep deprivation (8.5 min); melatonin was found to be non‐inferior in inducing sleep.
- Melatonin 5 mg is a safe, effective, and practical alternative to sleep deprivation for facilitating pediatric EEG recordings.

## 1 INTRODUCTION

Melatonin (5‐Methoxy‐N‐acetyltryptamine) is a hormone produced by the pineal gland that plays a key role in regulating circadian rhythms and the sleep–wake cycle. Due to its natural ability to promote sleep, it has been extensively used to address sleep disorders in both children and adults.^1^, ^2^ The safety and tolerability of melatonin have been well documented, and it does not significantly impact sleep macrostructure or epileptiform EEG activity.^3^, ^4^ Consequently, melatonin has become a possible strategy for sleep induction in clinical settings, particularly in pediatric and adult epilepsy centers.^5^ Typically, melatonin is administered in doses ranging from 5–10 mg/day.^6^ However, there are no specific guidelines for obtaining a sleep EEG, and there is no consensus on the optimal timing and dosing of melatonin for EEG recordings.

EEG recordings during sleep can be essential for some epilepsy diagnoses and for pediatric patients with cognitive disabilities. These recordings help minimize artifacts associated with patient cooperation and improve the diagnostic yield of the test by serving as an activating procedure that uncovers epileptic abnormalities.^7^, ^8^ Routine nap EEGs are generally performed during daytime hours, necessitating effective sleep induction strategies. While sleep deprivation can induce sleep, it often poses significant challenges and discomfort, especially for children facing neurodevelopmental disorders or behavioral issues.^8^ Historically, pharmacological agents like barbiturates, chlorpromazine, and chloral hydrate have been utilized to induce sleep. However, these substances can disrupt the macrostructure of sleep and affect EEG interpretations by distorting background activity or suppressing epileptiform abnormalities. Additionally, they may lead to prolonged sleepiness.^9^ In recent years, melatonin has garnered increasing interest as an alternative for nap EEG recordings.^9^, ^10^, ^11^, ^12^, ^13^, ^14^ Studies have documented melatonin's effectiveness in inducing sleep for EEG procedures, with a favorable safety profile and minimal impact on epileptiform activity.^15^ The absence of standardized guidelines regarding the timing and dosage of melatonin for sleep EEG recordings remains a significant gap in clinical practice. To address this, we have designed a randomized, crossover, non‐inferiority trial to assess the efficacy of a 5 mg melatonin solution in comparison to partial sleep deprivation alone for the induction and maintenance of sleep during nap EEG recordings.^16^ This manuscript presents the trial results and suggests a standardized hypno‐induction protocol that could be considered for implementation across pediatric neurophysiology centers.

## 2 MATERIALS AND METHODS

This study is a monocentric, randomized, crossover, non‐inferiority trial designed to compare the efficacy of melatonin 5 mg versus partial sleep deprivation for sleep induction in nap EEG among children and young adults with epilepsy or suspected epilepsy. Conducted at the Child Neurology and Psychiatry Unit of the IRCCS Mondino Foundation in Pavia, Italy, the trial was approved by the local Ethics Committee (Reference N°: P‐20200099096) and is registered at ClinicalTrials.gov (identifier: NCT05654415). Written informed consent was obtained from all participants or their guardians, with full information provided during a counseling session. Child neurologists and neurophysiologists involved in the study were trained in the relevant procedures and Good Clinical Practice guidelines.

### 2.1 Participants

**FIGURE 1:**
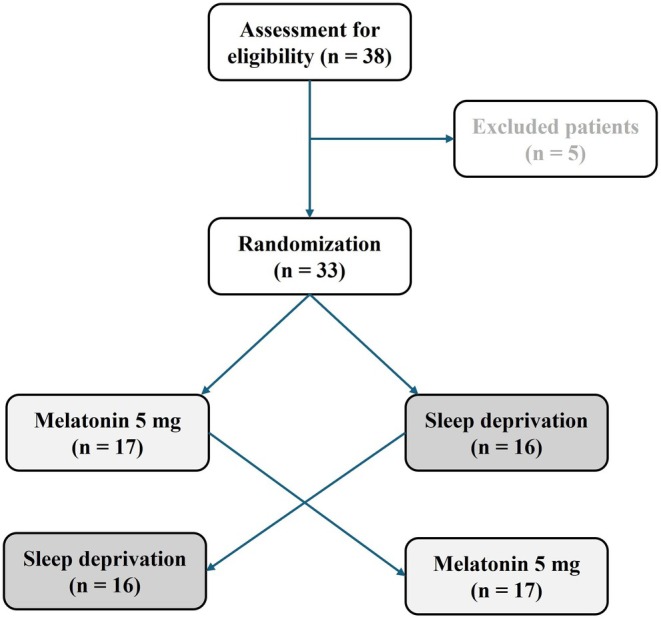
CONSORT flowchart for enrollment and randomization.

Participants were randomly allocated in a 1:1 ratio to receive either melatonin or partial sleep deprivation as the first intervention. Subjects in the first sequence (*n* = 16) received melatonin in the initial period and underwent sleep deprivation in the subsequent period, while those in the second sequence (*n* = 17) received the interventions in the reverse order. A total of 66 EEGs were obtained. The CONSORT flow diagram illustrating the study design is shown in Figure 1.

Eligible participants included pediatric patients aged 6 years and older who weighed ≥15 kg, had well‐controlled epilepsy or suspected epilepsy and/or EEG abnormalities, normal psychomotor development, stable seizure frequency, and stable antiseizure medication (ASM) treatment for at least 3 months prior to the study.^17^

Exclusion criteria included diagnoses of obstructive sleep apnea or other sleep disturbances, neurodevelopmental disorders, additional pharmacological therapies besides ASMs, use of hypnotics or stimulants, history of daily melatonin use, or any condition that might compromise study objectives.

### 2.2 Randomization, sleep induction, and EEG monitoring

Randomization was performed using R software (R Core Team, 2021) to generate random sequences for the crossover design. A computerized algorithm ensured random assignment to either the melatonin‐sleep deprivation or sleep deprivation‐melatonin sequence, without adjustments for demographic or clinical factors. The intervention consisted of administering a single oral dose of 5 mg of melatonin 30 min prior to the EEG recording. A fixed oral dose of 5 mg melatonin was selected for all participants, based on prior pediatric studies demonstrating efficacy and safety across a wide range of ages and weights,^10^, ^17^ as well as current clinical guidelines recommending 3–10 mg for children aged ≥12 months.

Partial sleep deprivation was induced by cutting the patient's usual nightly sleep duration by 50% the night prior to the EEG. Caregivers were contacted a week before the EEG to discuss sleep habits and ensure compliance with the sleep deprivation protocol. Participants were instructed to avoid napping on the morning of the examination and on their way to the hospital. Each EEG was conducted in a dark, quiet room at 1:30 p.m., and lasted 1 h. EEG recordings were made using a 10/20 electrode system, with polygraphic channels for ECG and respiration, and electromyogram activity recorded as appropriate for the patient's epileptic syndrome.

### 2.3 Outcomes

The primary outcome measure was sleep onset latency, which was defined as the time taken from full relaxation to the onset of non‐REM stage 2 sleep as indicated by the EEG.

Secondary outcome was the evaluation of procedure tolerability, based on the observation of drowsiness 2 h after the EEG recording.

### 2.4 Assay of melatonin and 6‐hydroxy‐melatonin in saliva samples

Salivary concentrations of melatonin and its main metabolite, 6‐hydroxy‐melatonin, were measured using a validated LC–MS/MS method. Oral fluid samples were collected 30 min after EEG recording and stored at −80°C until analysis. Detailed information on sample preparation and analytical settings is available in the Supplementary Methods section.

### 2.5 Sample size calculation and statistics

The sample size calculation, based on a non‐inferiority hypothesis for sleep onset latency, determined that a minimum of 60 EEG recordings (30 for each sequence) were required to achieve 80% power at a one‐sided significance level of 0.025. This calculation accounted for an expected difference in the lower 97.5% one‐sided confidence limit exceeding −3.0 min in sleep latency and was informed by prior studies reporting an average improvement of approximately 11 min with melatonin administration. Data were analyzed using descriptive statistics. Dichotomous variables were compared using chi‐square tests, while continuous variables were assessed using parametric or nonparametric tests depending on their distribution. An ANOVA for a crossover design was used to calculate the 97.5% lower confidence limit for the treatment difference between groups (SAS PROC MIXED).

Statistical significance was defined as *p* ≤ 0.05 for all inferential tests, and a 97.5% confidence interval for non‐inferiority was constructed. To account for the high variability in sleep latency, a dummy variable was included as a covariate in the ANOVA model. All analyses were performed using SAS 9.4 software (SAS Institute Inc., Cary, NC, USA). The analysis of the primary study variable, sleep latency, was conducted on both the Full Analysis Set (FAS), which included all treated patients, and the Per Protocol (PP) population, consisting of patients with data available from both study periods. This approach adhered to international guidelines for non‐inferiority studies, including ICH‐E9 (Statistical Principles for Clinical Trials, September 1998), the ICH E9 (R1) Addendum on Estimands and Sensitivity Analysis in Clinical Trials (July 2020), and the FDA's Non‐Inferiority Clinical Trials to Establish Effectiveness Guidance for Industry (November 2016). Given the high variability observed in sleep onset latency, the ANOVA model included a categorical variable to stratify latency times into distinct classes.

The classification variable for stratifying sleep onset latency was defined according to the following criteria: group 1: ≤5 min; group 2: >5 min and ≤10 min; group 3: >10 min and ≤20 min; group 4: >20 min and ≤30 min; group 5: >30 min. This stratification allowed for a more refined analysis of sleep latency, accounting for the observed variability across the study population.

## 3 RESULTS

### 3.1 Demographic characteristics

Of the 38 enrolled patients, 33 completed randomization and were included in the study. The participants were 14 males (42.4%) and 19 females (57.6%), with an average age of 14.5 years (15.07 years for males and 14.15 years for females). The demographic and clinical characteristics of the study population are summarized in Tables 1 and 2. Of the participants, 42.42% were diagnosed with self‐limited focal epilepsy, while 45.45% were diagnosed with generalized idiopathic epilepsy. The remaining 12.12% did not have an epilepsy diagnosis but exhibited non‐specific EEG abnormalities or other conditions such as migraine.

**TABLE 1:** Demographical characteristics of the 33 patients enrolled in the study.

Gender	Male (*n* = 14)	42.42%
	Female (*n* = 19)	27.57%
Age (years)	Male (M = 15.07)	SD = 3.91
	Female (M = 14.15)	SD = 3.74
Weight (kg)	Male (*n* = 13; M = 73.7)	SD = 11.95
	Female (*n* = 17; M = 56.7)	SD = 14. 05

*Note*: Demographical characteristics of the study sample.

Abbreviations: %, percentage; kg, kilograms; M, mean; *n*, number; SD, standard deviation.

**TABLE 2:** Clinical characteristics of the 33 patients enrolled in the study.

ASMs (*n*)	28	84.84%
Diagnosis	Self‐limited Focal epilepsy (*n* = 14)	42.42%
	Idiopathic Generalized epilepsy (*n* = 15)	45.45%
	EEG abnormalities (*n* = 3)	9.09%
	Migraine (*n* = 1)	3.03%
Number of ASMs (*n*)	0 (pt = 5)	15.15%
	1 (pt = 23)	69.69%
	2 (pt = 3)	9.09%
	3 (pt = 2)	6.06%
SLD Sleep latency (M)	8.30 min	SD = 6.42 min
Mel Sleep latency (M)	10.14 min	SD = 8.07 min
SLD Sleep stages (*n*)		
Stage I	*n* = 9	27.27%
Stage II	*n* = 13	39.40%
Stage III	*n* = 9	27.27%
Mel Sleep Stages (*n*)		
Stage I	*n* = 11	33.33%
Stage II	*n* = 14	42.43%
Stage III	*n* = 5	15.15%
Sleep failure (*n*)	SLD = 2	6.06%
	Mel = 3	9.09%
2H Drowsiness (*n*)	SLD = 4	12.12%
	Mel = 2	6.06%
Recorded seizures (*n*)	SLD = 1	3.03%
	Mel = 1	3.03%

*Note*: Clinical characteristics of the study sample.

Abbreviations: %, percentage; 2H Drowsiness, Drowsiness 2 h after EEG recording; ASMs, antiseizure medications; M, mean; Mel Sleep latency, time falling asleep with melatonin; Mel, Melatonin; min, minutes; *n*, number; SD, standard deviation; SLD Sleep latency, time falling asleep with sleep latency; SLD, Sleep deprivation; Sleep failure, patients who failed in falling asleep.

In terms of treatment, 28 participants (84.9%) were receiving at least one ASM. Among them, 69.69% were on monotherapy, while 15.15% were on polytherapy. Additionally, five participants were not receiving any treatment, including those without a formal epilepsy diagnosis.

Fifteen patients had experienced epileptic seizures in the year prior, but none in the 3 months leading up to the EEG recordings conducted as part of the protocol.

### 3.2 Sleep latency

**FIGURE 2:**
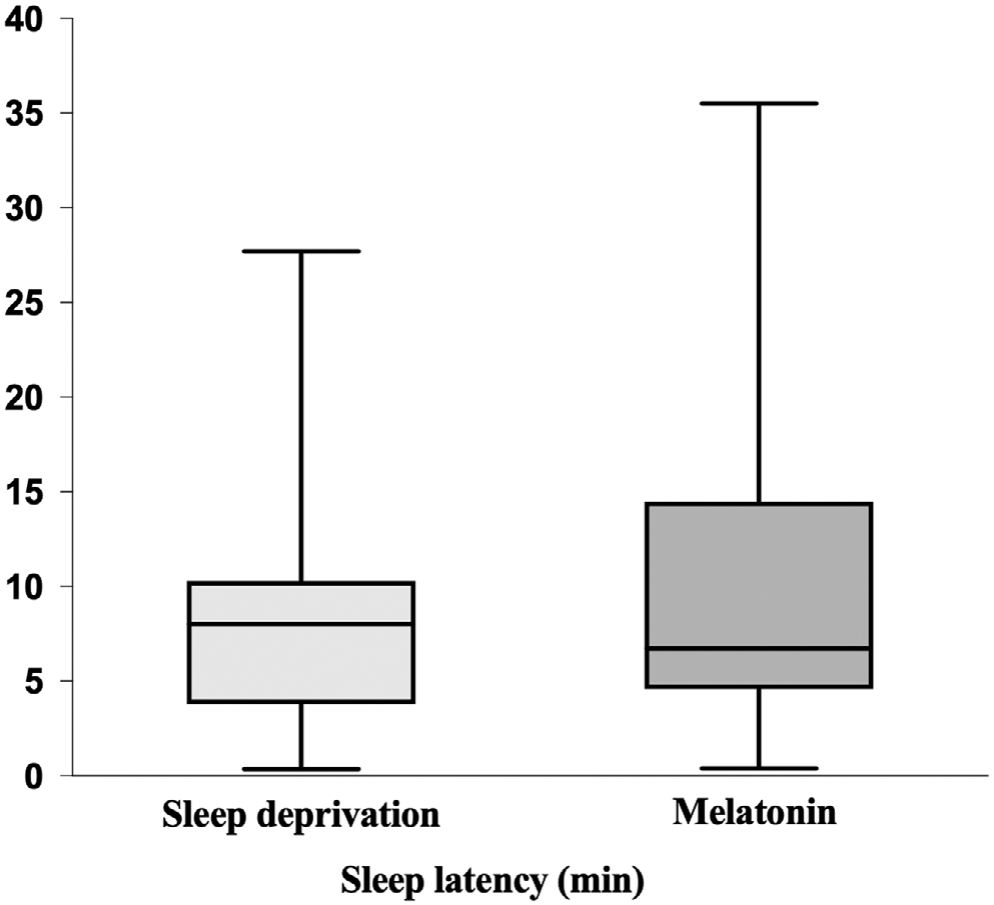
Box plot showing sleep latency in the melatonin 5 mg group and in the sleep deprivation group (*n* = 28 for each group). No statistically significant difference was observed (*p* = 0.5772), suggesting that, within this sample, melatonin 5 mg and sleep deprivation showed comparable effects on sleep latency.

All the 33 participants were randomly assigned to one of two groups: 5 mg of melatonin or partial sleep deprivation, as shown in Figure 1. Sleep onset times for each participant were recorded in minutes for both groups. Participants without data for both conditions were excluded from the PROC MIXED statistical analysis in SAS to calculate non‐inferiority. The analysis revealed that the average sleep onset time was 8.5 min for the partial sleep deprivation group and 10.1 min for the melatonin group, with a mean difference of 1.5 minutes in sleep onset times between the two groups (Figure 2). Analysis of sleep stages showed that: stage 1 was achieved by 9 patients after partial sleep deprivation and by 11 patients after melatonin administration, while stages 2–3 were reached by 22 patients after sleep deprivation and by 19 patients after melatonin administration. No patients achieved stage 4, as the recordings were obtained during afternoon naps. Two participants did not fall asleep after experiencing partial sleep deprivation, while three participants remained awake after taking 5 mg of melatonin.

The analysis of covariance for both the FAS and PP populations, conducted with stratification based on the classification variable for sleep onset latency, confirms the non‐inferiority of melatonin compared to sleep deprivation in sleep latency, with 97.5% lower confidence limits of −0.3707 and −0.3905 for the FAS and PP populations, respectively. Details are reported in Tables 3 and 4. In the FAS group (n = 28), the mean sleep latency in the melatonin condition was 10.1 ± 8.21 min, with a median of 6.7 min (range: 0.4–35.5 min). Following sleep deprivation, sleep latency was reduced, with a mean of 8.6 ± 6.61 min and a median of 8.0 min (range: 0.4–27.7 min). In the PP group (*n* = 26), sleep latency in the melatonin condition had a mean of 10.6 ± 8.27 min, with a median of 6.7 min (range: 3.4–35.5 min). Under sleep deprivation, sleep latency was slightly lower, with a mean of 9.1 ± 6.53 min and a median of 8.1 min (range: 0.8–27.7 min). The carryover effect, assessed through the SEQUENCE factor in the mixed model, was not statistically significant (*p* = 0.33).

**TABLE 3:** Sleep Latency in the FAS set. Non‐inferiority: Difference between treatments −0.35; non‐inferiority limit −0.3707/+∞.

Treatments	Sleep latency category (min)	*N*	Mean	SD	Median	Min	Max
Melatonin	≤5	9	3.8	1.37	4.1	0.4	5.0
	5.1–10	10	6.9	1.25	6.7	5.2	9.4
	10.1–20	5	15.2	3.78	15.0	10.2	19.3
	20.1–30	3	22.7	2.46	21.7	21.0	25.6
	>30.1	1	35.5		35.5	35.5	35.5
Sleep latency	≤5	12	3.2	1.49	3.5	0.4	4.8
	5.1–10	9	8.3	1.11	8.1	6.1	10.0
	10.1–20	5	15.0	3.09	15.4	10.4	18.4
	20.1–30	2	25.0	3.78	25.0	22.4	27.7

**TABLE 4:** Sleep Latency in the PP set. Non‐inferiority: Difference between treatments −0.37; non‐inferiority limit −0.3905/+∞.

Treatments	Sleep latency category (min)	*N*	Mean	SD	Median	Min	Max
Melatonin	≤5	8	4.2	0.59	4.2	3.4	5.0
	5.1–10	9	6.8	1.33	6.6	5.2	9.4
	10.1–20	5	15.2	3.78	15.0	10.2	19.3
	20.1–30	3	22.7	2.46	21.7	21.0	25.6
	>30.1	1	35.5		35.5	35.5	35.5
Sleep latency	≤5	10	3.6	1.25	3.9	0.8	4.8
	5.1–10	9	8.3	1.11	8.1	6.1	10.0
	10.1–20	5	15.0	3.09	15.4	10.4	18.4
	20.1–30	2	25.0	3.78	25.0	22.4	27.7

### 3.3 Procedure tolerability

Approximately 2 hours after the Nap EEG recording, subjective drowsiness was assessed. In total, four patients in the sleep deprivation group and one patient in the melatonin group reported drowsiness.

Additionally, an epileptic seizure was recorded during EEG video monitoring in both groups of the study during NREM sleep.

### 3.4 Method validation and salivary levels of melatonin and 6‐hydroxy‐melatonin

**FIGURE 3:**
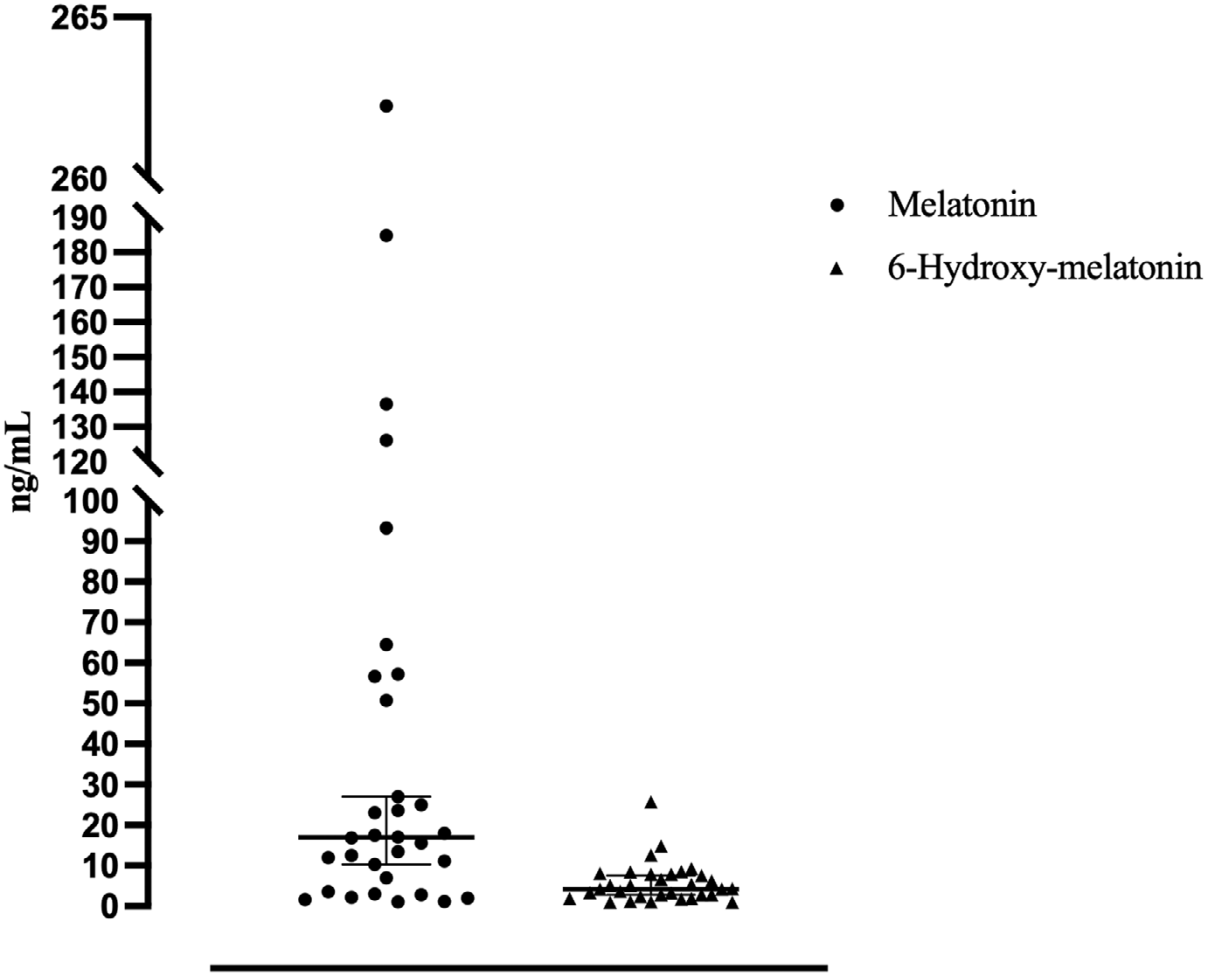
Distribution of salivary melatonin and 6‐hydroxy‐melatonin levels in 32 patients with epilepsy.

The analytical method showed high reliability, with precision <13% for melatonin and <15% for 6‐hydroxy‐melatonin, accuracy within 85%–112%, and strong linearity across the tested concentration ranges. Melatonin exhibited excellent stability under short‐term and long‐term storage, while 6‐hydroxy‐melatonin showed partial instability after repeated freeze–thaw cycles. The results obtained are in accordance with the validation guidelines for the bioanalytical methods from the European Medicines Agency.^18^ In participants receiving melatonin 5 mg, mean salivary concentrations were 41 ng/mL for melatonin and 6 ng/mL for 6‐hydroxy‐melatonin (Figure 3). In the sleep deprivation group, both analytes were below the lower limit of quantification. Full details on method validation and stability data are reported in the Supplementary Results section.

## 4 DISCUSSION

Sleep EEG recording enhances diagnostic accuracy and facilitates the appropriate management of pediatric and cognitively impaired patients, since it allows high‐quality recordings and helps detect epileptiform abnormalities when epilepsy is known or suspected.^7^, ^8^ However, achieving sleep during EEG recordings can be challenging, as most outpatient EEGs are performed during the daytime. Despite the critical need for sleep induction approaches during EEG recordings, there is currently no consensus—either internationally or nationally—on standardized strategies for facilitating sleep. The most common techniques for inducing sleep during EEG are sleep deprivation and melatonin administration. Sleep deprivation, while often preferred, can be highly stressful for patients and their families, thereby reducing compliance and tolerability. Informal feedback from caregivers highlighted that enforcing partial sleep deprivation was often challenging, leading to stress, disrupted routines, and difficulty keeping children awake the night before EEGs. In contrast, melatonin administration was described as simple and straightforward, requiring minimal effort from families, which supports its greater tolerability.

However, there is no agreement regarding the dosage, timing, or specific administration protocols. Studies in this area remain highly heterogeneous, with limited evidence favoring one approach over another due to observational or retrospective designs, lack of randomization, and variability in populations and protocols.^5^, ^9^, ^10^, ^11^, ^12^, ^13^, ^14^

In this context, our study was designed and conducted prospectively to evaluate the effectiveness and non‐inferiority of a fixed dose of 5 mg melatonin compared to sleep deprivation alone for inducing sleep during EEG in routine clinical practice in patients aged 6 years and older (>15 kg). As per our primary outcome measure, our results demonstrated that administering a standard dose of 5 mg melatonin is non‐inferior to 50% physiological sleep deprivation in terms of sleep onset time and the number of successfully completed EEG recordings. Additionally, the frequency of electroclinical episodes captured during EEG was identical in both groups. This finding supports the conclusion that melatonin, administered at a moderate fixed dose, can serve as an effective sleep‐inducing agent in the EEG laboratory setting, without causing significant drowsiness or other side effects 2 h after administration.

Melatonin and its metabolite salivary quantitation confirmed adequate systemic absorption following oral administration.

Our standardized 5 mg melatonin protocol could be readily implemented across pediatric neurophysiology centers. Its simplicity—single oral administration 30 min prior to EEG—requires minimal training and resources, making it feasible even in centers with high patient volume. This approach is particularly advantageous for populations in whom sleep deprivation is challenging, such as children with neurodevelopmental disorders, behavioral difficulties, or complex family circumstances.

We hypothesize that adopting this melatonin protocol could maximize the number of successful sleep EEG recordings while reducing stress for families and minimizing the economic burden associated with repeated examinations due to incomplete electroclinical information.

Based on the results of our prospective, randomized study, this melatonin administration protocol appears to be a simple and well‐tolerated approach that could potentially be shared and considered for implementation in pediatric neurophysiology centers to enhance clinical efficiency while maintaining high‐quality diagnostic outcomes.

## 5 LIMITATIONS

This study has several limitations. First, its single‐center design may affect the generalizability of the results to other clinical settings. Second, although the sample size was adequate to assess non‐inferiority for the primary endpoint, it may limit the power for secondary outcomes and subgroup analyses. The open‐label design, inherent to the comparison between pharmacological intervention and sleep deprivation, precluded double‐blinding; however, EEG interpretation was performed by expert epileptologists blinded to the intervention. Furthermore, the protocol evaluated a fixed 5 mg dose of melatonin in children aged ≥6 years, which may restrict applicability to broader pediatric populations, including younger children and other dose regimens.

## 6 CONCLUSION

This randomized, crossover non‐inferiority trial demonstrates that the administration of a single 5 mg dose of melatonin is as effective as partial sleep deprivation in inducing sleep for nap EEG recordings in pediatric patients with epilepsy or suspected epilepsy.

These findings support the use of melatonin as a practical, well‐tolerated, and non‐invasive alternative to sleep deprivation during EEG sleep induction in clinical neurophysiology. Given its favorable safety profile and minimal impact on EEG quality, melatonin may be especially valuable in patients for whom sleep deprivation is contraindicated or difficult to implement, such as those with neurodevelopmental disorders.

This study contributes to filling a significant gap in clinical practice by proposing a standardized hypno‐induction protocol with melatonin for sleep EEGs. Further multicenter studies are warranted to validate these findings and develop evidence‐based guidelines for the routine use of melatonin in pediatric EEG settings.

## AUTHOR CONTRIBUTIONS

Conceptualization: VDG, CV, and VF; methodology: VDG, CV, MC, and GF; data curation: MC, CAQ, FF, LP, GP, and MP; writing—original draft preparation: VDG, CV, LP, and VF; writing—review and editing. All authors have read and agreed to the published VDG, CV, MC, CAQ, FF, LP, GF, GP, MP, and VF. All the authors approved the current version of the manuscript.

## FUNDING INFORMATION

This research was funded by a grant from the Italian Ministry of Health (Ricerca Corrente 2024–25) and by the Italian Ministry of Health through the “5 × 1000” program (IRCCS Mondino Foundation, Pavia, Italy), in partial fulfillment of the project.

## CONFLICT OF INTEREST STATEMENT

VDG received speaker and/or consultancy fees from Jazz Pharma, Orion, Longboard, Dr. Schar, and Nutricia. The remaining authors have no conflict of interest to declare. We confirm that we have read the Journal's position on issues involved in ethical publication and affirm that this report is consistent with those guidelines.

## ETHICS STATEMENT

The study was conducted in accordance with the Declaration of Helsinki and was approved by the Ethics Committee of IRCCS San Matteo Hospital, Pavia (protocol code 20200099096).

## INFORMED CONSENT

Written informed consent was obtained from all the subjects/parents of all the subjects involved in the study.

## Supporting information


Appendix S1:

## Data Availability

The authors are available to share the raw CRF data upon reasonable request.
