# Supplementary material for: A prospective randomized crossover trial investigating melatonin versus sleep deprivation for sleep induction in nap electroencephalography
Source: Epilepsia Open. 2025 Nov 10;11(1):136–45. doi: 10.1002/epi4.70169 (PMC12903794; doi:10.1002/epi4.70169)
Supplement: Supplementary file 1 — Appendix S1: [file EPI4-11-136-s001.docx]

**Supplementary Methods**

**LC-MS/MS assay of melatonin and 6-hydroxy-melatonin**

This document provides the complete description of the analytical procedures used for the quantification of melatonin and its metabolite, 6-hydroxy-melatonin, in saliva samples using a validated liquid chromatography coupled to tandem mass spectrometry (LC-MS/MS) method. The detailed methodology, including sample preparation, chromatographic conditions, calibration parameters, and validation results, is reported below.

Salivary concentrations of melatonin and its main metabolite, 6-hydroxy-melatonin, were measured using a validated LC-MS/MS method. Oral fluid samples were obtained via passive drooling and collected 30 minutes after the EEG. All samples were stored at −80°C until analysis. To prepare the samples for LC-MS/MS analysis, 50 μL of saliva were mixed with 5 μL of an internal standard solution (315 ng/mL of melatonin-*d_4_* in water) and 5 μL of standard solutions at concentrations of 10, 60, 150, 300, 750, 1500, and 3000 ng/mL for melatonin, and 10, 20, 50, 100, 250, 500, and 1000 ng/mL for 6-hydroxy-melatonin, both in water. Additionally, 60 μL of a precipitating agent (0.2 M ZnSO_4_ and methanol 30:70 v/v) were added. For quality control samples, 50 μL of saliva were mixed with 5 μL of the internal standard solution and 5 μL of standard solutions at concentrations of 15, 1050, and 2250 ng/mL for melatonin, and 15, 350, and 750 ng/mL for 6-hydroxy-melatonin, along with 60 μL of the precipitating agent. For the unknown samples, 50 μL of saliva were combined with 5 μL of the internal standard solution and 65 μL of the precipitating agent. After vortexing, the solution was centrifuged for 10 minutes at 4°C and 17,000× g, and then 40 μL of the supernatant was injected into the LC-MS/MS system.

The analytical procedure was performed by using an LC-MS/MS system consisting of an ExionLC 100 HPLC integrated system coupled to a 3200 QTRAP® triple-quadrupole mass spectrometer (Applied Biosystems Sciex, Darmstadt, Germany) equipped with an electrospray ionization source operating in positive mode. Data acquisition was performed using multiple reaction monitoring (MRM) with the following transitions: m/z 233.2 → 174.2 (quantifier transition) and 159.2 (qualifier transition) for melatonin, m/z 249.2 → 190.2 (quantifier transition) and 158.1 (qualifier transition) for 6-hydroxy-melatonin, and m/z 237.2 → 178.3 (quantifier transition) and 163.2 (qualifier transition) for the internal standard melatonin-*d_4_*. The purification and enrichment of the sample were carried out online with a POROS R1 column (2.1 × 30 mm i.d., 20 μm, Thermo Fisher Scientific, Waltham, Massachusetts, USA). For the separation of analytes, a monolithic C18 column (Onyx, 100 × 3 mm i.d., Phenomenex, Bologna, Italy) was employed, with the column temperature set at 25°C. The mobile phase consisted of two components, water with 0.1% formic acid (A) and methanol with 0.1% formic acid (B), and the analysis was done using a gradient as follows: 98% A and 2% B for one minute at 1 mL/min, from 2% to 100% B from 1 to 2 minutes at 0.6 mL/min, 100% B from 2 to 11 minutes at 0.6 mL/min, and back to 98% A and 2% B from 11.05 to 14 minutes at 0.6 mL/min. The chromatographic conditions employed resulted in melatonin and the internal standard eluting at 3.82 minutes, whereas 6-hydroxy-melatonin eluted at 3.68 minutes.

**Supplementary Results**

**Method validation**

This section provides the complete description of the method validation parameters for melatonin and 6-hydroxy-melatonin quantification using LC-MS/MS, including precision, accuracy, calibration curves, recovery rates, matrix effects, stability, and salivary concentration data.

Precision and accuracy values for melatonin and 6-hydroxy-melatonin were assessed on both the same day and across three different days. The precision of QCs for melatonin and 6-hydroxy-melatonin within and across days was below 6.87% and 11.16% respectively. At the lower limit of quantification (LLOQ), the precision within and across days was below 13.09% for melatonin and 15.46% for 6-hydroxy-melatonin. Intra-day and interday-accuracy ranged between 89.20% and 111.21% for melatonin, and 85.40% and 112.26% for 6-hydroxy-melatonin. LLOQ was set at 1 ng/mL for both analytes, while the LOD was established at 0.1 ng/mL for melatonin and 0.5 ng/mL for 6-hydroxy-melatonin. Calibration curves exhibited linearity across the concentration ranges of 1-300 ng/mL for melatonin and 1-100 ng/mL for 6-hydroxy-melatonin. The coefficients of correlation exceeded 0.999 for both analytes, and the average slopes observed over five different days were 0.041 and 0.011 for melatonin and 6-hydroxy-melatonin, respectively. Mean recovery rates at low, medium, and high QC levels were 117.03%, 106.77%, and 98.16% for melatonin, and 117.24%, 104.88%, and 102.21% for 6-hydroxy-melatonin. Furthermore, the impact of matrix effects was not significant, with precision values below 7.73% and 14.61% for melatonin and 6-hydroxy-melatonin, respectively, while accuracy fell within the range of 86.44% to 97.88% for melatonin and 89.11% to 114.08% for 6-hydroxy-melatonin. No interference peaks were present at the retention times of melatonin, 6-hydroxy-melatonin, and IS in saliva samples collected from six different healthy individuals and carry-over effects from blank injections following the highest calibrator (300 ng/mL for melatonin and 100 ng/mL for 6-hydroxy-melatonin) were absent for all analytes. The reproducibility of reinjection samples demonstrated precision below 9.25% for both analytes, while accuracy ranged from 96.57% to 101.07% for melatonin and from 92.53% to 108.83% for 6-hydroxy-melatonin. These results are in accordance with the validation guidelines for the bioanalytical methods from the European Medicines Agency^18^. Melatonin levels in saliva samples exhibited stability under various conditions: at room temperature for up to 24 hours and after being in the autosampler at room temperature for 24 hours, as well as when stored at -80 °C for 7 months and after three cycles of freezing at -80 °C for 1 hour followed by thawing at room temperature for 15 minutes. In contrast, 6-hydroxy-melatonin in saliva samples showed stability when stored at 4 °C for 1 hour, after being in the autosampler for 24 hours at room temperature and when stored at -80 °C for 3 days. However, it was noted to be unstable after undergoing freeze-thaw cycles.
